# Methyl [hydr­oxy(phen­yl)phosphono­meth­yl]phospho­nate methanol solvate

**DOI:** 10.1107/S160053680802285X

**Published:** 2008-09-06

**Authors:** Nathalie Dupont, Pascal Retailleau, Evelyne Migianu-Griffoni, Carole Barbey

**Affiliations:** a Laboratoire de Biophysique Moléculaire, Cellulaire et Tissulaire, UMR 7033 CNRS, UFR–SMBH Université Paris-Nord, 74 rue M. Cachin, 93017 Bobigny Cedex, France; b Service de Cristallochimie, Institut de Chimie des Substances Naturelles, CNRS, 1 Avenue de la Terrasse, 91198 Gif sur-Yvette Cedex, France

## Abstract

The title compound, C_8_H_12_O_7_P_2_·CH_4_O, is a monoesterified bis­phospho­nate (or 1-hydroxy­methyl­ene-1,1-bis­phospho­nic acid). These synthetic compounds are widely used in medicine to inhibit bone resorption in diseases like osteoporosis, and are characterized by a stable P—C—P group and are thus analogs of inorganic pyrophosphate. By masking one or several ionizable groups, introduced as phosphono­ester, it was anti­cipated the formation of prodrugs with higher lipophilicity that could facilitate the drug delivery and metabolization. Mol­ecules are paired by inter­molecular hydrogen bonds involving the phospho­nic groups. In addition, dimers are connected side-by-side, building infinite ribbons along the *a*-axis direction; these ribbons are cross-linked perpendicularly along the *b*-axis direction *via* a methanol solvent mol­ecule (disordered over two sites with occupancy factors *ca* 0.6 and 0.4), forming an extended inter­molecular hydrogen-bonded network. The H atoms of the methyl group in the main molecule are disordered equally over two positions.

## Related literature

For related literature, see: Barbey *et al.* (2003 ▶), Migianu *et al.* (2005 ▶), Fleisch (1998 ▶, 2002 ▶); Clezardin *et al.* (2003 ▶); Green & Clezardin (2002 ▶); Lecouvey *et al.* (2003*a* ▶,*b* ▶); Vepsalainen (2002 ▶).
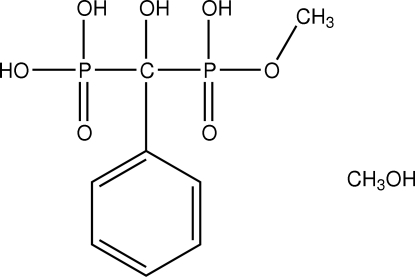


## Experimental

### 

#### Crystal data

C_8_H_12_O_7_P_2_·CH_4_O
*M* *_r_* = 314.16
Monoclinic, 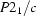

*a* = 6.3085 (5) Å
*b* = 6.9871 (6) Å
*c* = 28.147 (2) Å
β = 92.654 (3)°
*V* = 1239.34 (17) Å^3^
*Z* = 4
Mo *K*α radiation
μ = 0.39 mm^−1^
*T* = 293 (2) K
0.30 × 0.20 × 0.20 mm

#### Data collection

Nonius KappaCCD diffractometer
Absorption correction: multi-scan (*SCALEPACK*; Otwinowski & Minor, 1997 ▶) *T*_min_ = 0.847, *T*_max_ = 0.929
3837 measured reflections
2375 independent reflections
1943 reflections with *I* > 2σ(*I*)
*R*_int_ = 0.033

#### Refinement

*R*[*F* ^2^ > 2σ(*F* ^2^)] = 0.057
*wR*(*F* ^2^) = 0.141
*S* = 1.08
2375 reflections
191 parameters
32 restraints
H-atom parameters constrained
Δρ_max_ = 0.56 e Å^−3^
Δρ_min_ = −0.44 e Å^−3^

### 

Data collection: *COLLECT* (Hooft, 1998 ▶); cell refinement: *HKL* (Otwinowski & Minor, 1997 ▶); data reduction: *COLLECT*; program(s) used to solve structure: *SHELXS97* (Sheldrick, 2008 ▶); program(s) used to refine structure: *SHELXL97* (Sheldrick, 2008 ▶); molecular graphics: *ORTEP-3 for Windows* (Farrugia, 1997 ▶) and *PLATON* (Spek (2003 ▶); software used to prepare material for publication: *WinGX* (Farrugia, 1999 ▶) and *CrystalBuilder* (Welter, 2006 ▶).

## Supplementary Material

Crystal structure: contains datablocks global, I. DOI: 10.1107/S160053680802285X/pk2106sup1.cif

Structure factors: contains datablocks I. DOI: 10.1107/S160053680802285X/pk2106Isup2.hkl

Additional supplementary materials:  crystallographic information; 3D view; checkCIF report

## Figures and Tables

**Table 1:** Hydrogen-bond geometry (Å, °)

*D*—H⋯*A*	*D*—H	H⋯*A*	*D*⋯*A*	*D*—H⋯*A*
O13—H13⋯O21^i^	0.82	1.75	2.548 (4)	163
O11—H11⋯O22^ii^	0.82	1.72	2.525 (4)	169
O21—H21⋯O62^iii^	0.82	1.89	2.548 (5)	136
O7—H7⋯O12^iv^	0.82	1.86	2.658 (3)	164

Symmetry codes: (i) 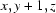; (ii) 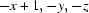; (iii) 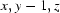; (iv) 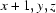.

**Fig. 1.**
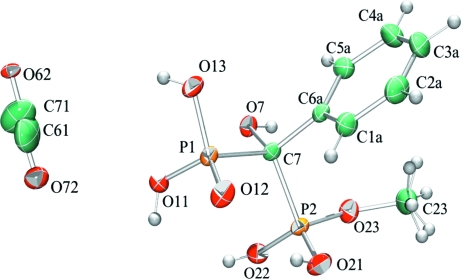
Molecular view of the title compound. Displacement ellipsoids are drawn at the 40 % probability level.

**Fig. 2.**
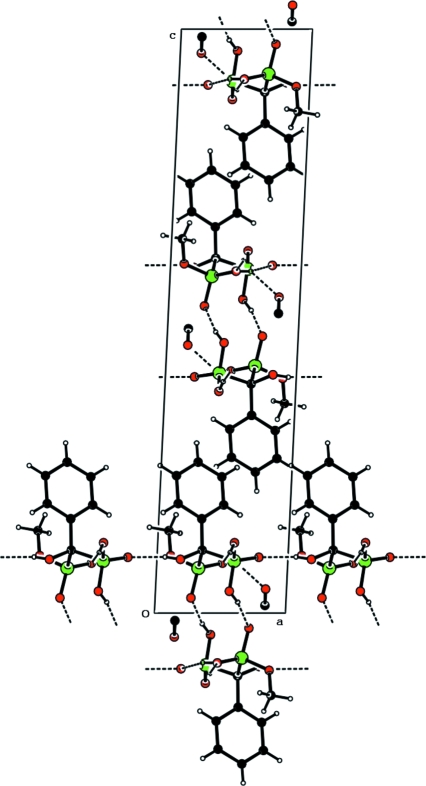
Partial packing view, projected along the b axis, showing the formation of the bisphosphonate dimers and the two dimensional network. H-bonds are represented as dashed lines. (PLUTO diagram from PLATON (Spek, 2003))

**Fig. 3.**
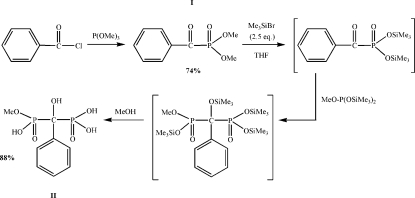
Scheme 2. Chemical pathway of the formation of title compound (II)

## supplementary crystallographic information

### Comment

The title compound, C_8_H_12_O_7_P_2_, is a potential prodrug of the corresponding 1-hydroxymethylene-1,1-bisphosphonate (HMBP). This family of molecules has recently become very interesting owing to their biological properties and medical applications. Indeed, they are used in nuclear medicine, in treatment of bone diseases (Paget's disease, osteoporosis) and as adjuvant in the treatment of some cancers (e.g. breast, prostate) due to their antiproliferative properties (Fleisch, 2002; Green & Clezardin, 2002; Clezardin *et al.*, 2003). However, HMBPs show a low intestinal absorption because of their high hydrophilicity and complexing power towards divalent cations of the organism. Moreover, they induce some secondary effects such as gastric and intestinal problems and osteonecrosis of the jaw-bone. To circumvent to these drawbacks, a prodrug strategy was considered that would deliver bisphosphonates with an improved gastrointestinal absorption (Vepsalainen, 2002). The approach in our laboratory consists of modifying the phosphonic acid functionality itself, by introducing an ester group (Lecouvey *et al.*, 2003*a*,*b*). Thus, by masking the negative charges of HMBPs with suitable bioreversible substituents, the lipophilicity of bisphosphonates could be enhanced and the complexation with divalent cations decreased. Bisphosphonate prodrugs should then release bisphosphonic acids *via* enzymatic and/or chemical hydrolysis. Among these synthesized prodrugs, the title compound is a monoesterified version for which we report herein the crystal structure determination (Fig. 1). The crystal structure consists of layers of hydrophobic regions that enclose the phenyl rings and polar regions where bisphosphonate groups are linked as pairs and a disordered methanol molecule takes part in the crystal cohesion (Fig. 2).

### Experimental

Synthesis of the α-ketophosphonate dimethyl ester (I): benzoyl chloride (5.8 ml, 50 mmol) was added dropwise at -10°C under argon to trimethylphosphite (5.9 ml, 50 mmol). The reaction mixture was then stirred at room temperature for 2 h (the end of the reaction was monitored by ^31^P {1H} NMR or IR spectroscopy). The crude product was purified by distillation under reduced pressure to furnish the desired α-ketophosphonate dimethyl ester with 74% yield (Migianu *et al.*, 2005, compound 2 d).

Synthesis of [hydroxy-(hydroxy-methoxy-phosphoryl)-phenyl-methyl]-phosphonic acid (II): To the α-ketophosphonate dimethyl ester (1.07 g, 5 mmol) in 4 ml of distilled THF at 0°C under argon was added dropwise trimethylsilyl bromide (1.65 ml, 12.5 mmol). The reaction was exothermic and the temperature had to be maintained below 10°C during the addition. The reaction mixture was stirred at room temperature for 5 h (the end of the reaction was monitored by ^31^P {1H} NMR) and evaporation of volatile fractions (0.01 Torr) at 50°C gave bis(silylated) α-ketophosphonate. Methyl bis(trimethylsilyl) phosphite (1.2 g, 5 mmol) was then added dropwise at 0°C under argon. The reaction mixture was stirred overnight at room temperature and methanolysis for two hours led to the expected 1-hydroxymethylene-1,1- bisphosphonate monomethyl ester. After reduced pressure evaporation of volatile fractions, the crude compound was purified by precipitation in methanol and obtained with 88% yield (Scheme 2, Migianu *et al.*, 2005).

Crystallization of monomethylester II was by slow evaporation at room temperature from a concentrated methanol/ water (9/1) solution to give colorless crystals with max. size 0.3 mm, suitable for diffraction.

### Refinement

All H atoms attached to C or O atoms were fixed geometrically and treated as riding with C—H = 0.93 Å (aromatic) or 0.96 Å (methylene) and O—H = 0.82 Å (hydroxyl) with *U*_iso_(H) = 1.2*U*_eq_(C) (aromatic) or 1.5*U*_eq_(C) and 1.5*U*_eq_(O) for others. The methyl group was refined as idealized disordered one with two positions rotated from each other by 60 degrees. Each of the P2—O21 and P2—O22 bonds seems to be a mixture of single and double bonds, so the disordered hydroxyl group bound to P2 was modeled as constrained hydrogen with a site occupation factors of 0.5 on each site. The solvent molecule is a disordered one with two alternative conformations on a single site. H atoms of this disordered methanol molecule are intentionaly not included because they are very difficult to position accurately.

### Crystal data

**Table:**

C_8_H_12_O_7_P_2_·CH_4_O	*Z* = 4
*M**_r_* = 314.16	*F*(000) = 656
Monoclinic, *P*2_1_/*c*	*D*_x_ = 1.684 Mg m^−^^3^
Hall symbol: -P 2ybc	Mo *K*α radiation, λ = 0.71073 Å
*a* = 6.3085 (5) Å	θ = 0.4–26.0°
*b* = 6.9871 (6) Å	µ = 0.39 mm^−^^1^
*c* = 28.147 (2) Å	*T* = 293 K
β = 92.654 (3)°	Parallelepiped, colourless
*V* = 1239.34 (17) Å^3^	0.30 × 0.20 × 0.20 mm

### Data collection

**Table:**

Nonius KappaCCD diffractometer	2375 independent reflections
Radiation source: fine-focus sealed tube	1943 reflections with *I* > 2σ(*I*)
graphite	*R*_int_ = 0.033
Detector resolution: 9 pixels mm^-1^	θ_max_ = 25.9°, θ_min_ = 3.3°
φ and ω scans	*h* = −7→7
Absorption correction: multi-scan (SCALEPACK; Otwinowski & Minor, 1997)	*k* = −7→8
*T*_min_ = 0.847, *T*_max_ = 0.929	*l* = −34→34
3837 measured reflections	

### Refinement

**Table:**

Refinement on *F*^2^	Primary atom site location: structure-invariant direct methods
Least-squares matrix: full	Secondary atom site location: difference Fourier map
*R*[*F*^2^ > 2σ(*F*^2^)] = 0.057	Hydrogen site location: inferred from neighbouring sites
*wR*(*F*^2^) = 0.142	H-atom parameters constrained
*S* = 1.08	*w* = 1/[σ^2^(*F*_o_^2^) + (0.0456*P*)^2^ + 3.3095*P*] where *P* = (*F*_o_^2^ + 2*F*_c_^2^)/3
2375 reflections	(Δ/σ)_max_ < 0.001
191 parameters	Δρ_max_ = 0.56 e Å^−^^3^
32 restraints	Δρ_min_ = −0.44 e Å^−^^3^

### Special details

**Table:**

Geometry. All e.s.d.'s (except the e.s.d. in the dihedral angle between two l.s. planes) are estimated using the full covariance matrix. The cell e.s.d.'s are taken into account individually in the estimation of e.s.d.'s in distances, angles and torsion angles; correlations between e.s.d.'s in cell parameters are only used when they are defined by crystal symmetry. An approximate (isotropic) treatment of cell e.s.d.'s is used for estimating e.s.d.'s involving l.s. planes.
Refinement. Refinement of *F*^2^ against ALL reflections. The weighted *R*-factor *wR* and goodness of fit *S* are based on *F*^2^, conventional *R*-factors *R* are based on *F*, with *F* set to zero for negative *F*^2^. The threshold expression of *F*^2^ > σ(*F*^2^) is used only for calculating *R*-factors(gt) *etc*. and is not relevant to the choice of reflections for refinement. *R*-factors based on *F*^2^ are statistically about twice as large as those based on *F*, and *R*- factors based on ALL data will be even larger.

### Fractional atomic coordinates and isotropic or equivalent isotropic displacement parameters (Å^2^)

**Table:**

	*x*	*y*	*z*	*U*_iso_*/*U*_eq_	Occ. (<1)
P1	0.41077 (14)	0.23600 (13)	0.08924 (3)	0.0240 (3)	
O11	0.4358 (4)	0.3030 (4)	0.03727 (9)	0.0317 (6)	
H11	0.3700	0.2305	0.0190	0.047*	
O12	0.2224 (4)	0.1143 (4)	0.09548 (10)	0.0371 (7)	
O13	0.4134 (4)	0.4188 (4)	0.12031 (9)	0.0340 (6)	
H13	0.4428	0.5116	0.1040	0.051*	
P2	0.68094 (15)	−0.12727 (14)	0.07691 (3)	0.0273 (3)	
O21	0.4976 (4)	−0.2548 (4)	0.08552 (11)	0.0410 (7)	
H21	0.3883	−0.1912	0.0851	0.061*	0.50
O22	0.7272 (5)	−0.0849 (4)	0.02594 (9)	0.0394 (7)	
H22	0.6239	−0.0328	0.0127	0.059*	0.50
O23	0.8962 (4)	−0.2125 (4)	0.09820 (10)	0.0377 (7)	
C23	0.9189 (8)	−0.3297 (7)	0.14094 (17)	0.0532 (12)	
H23A	1.0653	−0.3639	0.1466	0.080*	0.50
H23B	0.8352	−0.4438	0.1368	0.080*	0.50
H23C	0.8711	−0.2588	0.1676	0.080*	0.50
H23D	0.7825	−0.3471	0.1541	0.080*	0.50
H23E	1.0126	−0.2672	0.1639	0.080*	0.50
H23F	0.9766	−0.4522	0.1330	0.080*	0.50
C1A	0.5094 (7)	−0.0159 (6)	0.18502 (14)	0.0360 (9)	
H1A	0.3840	−0.0484	0.1683	0.043*	
C2A	0.5336 (8)	−0.0566 (7)	0.23324 (15)	0.0455 (11)	
H2A	0.4235	−0.1146	0.2487	0.055*	
C3A	0.7199 (8)	−0.0115 (7)	0.25829 (14)	0.0464 (11)	
H3A	0.7371	−0.0416	0.2904	0.056*	
C4A	0.8802 (7)	0.0784 (7)	0.23536 (15)	0.0454 (11)	
H4A	1.0056	0.1096	0.2523	0.054*	
C5A	0.8572 (6)	0.1230 (6)	0.18731 (13)	0.0341 (9)	
H5A	0.9657	0.1861	0.1724	0.041*	
C6A	0.6723 (6)	0.0736 (5)	0.16151 (12)	0.0259 (8)	
C7	0.6573 (5)	0.1066 (5)	0.10757 (12)	0.0223 (7)	
O7	0.8204 (4)	0.2294 (4)	0.09186 (8)	0.0267 (6)	
H7	0.9357	0.1761	0.0958	0.040*	
O62	0.1603 (6)	0.8597 (7)	0.04096 (16)	0.0193 (14)	0.559 (10)
C61	0.159 (5)	0.695 (3)	0.0135 (8)	0.072 (6)	0.559 (10)
O72	0.1408 (11)	0.5812 (12)	−0.0056 (3)	0.037 (2)	0.441 (10)
C71	0.167 (5)	0.754 (4)	0.0172 (13)	0.067 (7)	0.441 (10)

### Atomic displacement parameters (Å^2^)

**Table:**

	*U*^11^	*U*^22^	*U*^33^	*U*^12^	*U*^13^	*U*^23^
P1	0.0226 (5)	0.0195 (5)	0.0300 (5)	0.0013 (4)	0.0009 (3)	0.0004 (4)
O11	0.0375 (14)	0.0296 (14)	0.0274 (13)	0.0005 (12)	−0.0040 (10)	−0.0004 (11)
O12	0.0213 (13)	0.0321 (15)	0.0580 (18)	−0.0016 (12)	0.0036 (11)	0.0031 (14)
O13	0.0476 (16)	0.0214 (14)	0.0335 (14)	0.0038 (12)	0.0075 (12)	−0.0028 (11)
P2	0.0311 (5)	0.0191 (5)	0.0314 (5)	0.0023 (4)	−0.0027 (4)	−0.0039 (4)
O21	0.0399 (16)	0.0255 (14)	0.0571 (19)	−0.0008 (13)	−0.0027 (13)	−0.0066 (13)
O22	0.0545 (17)	0.0334 (16)	0.0300 (14)	0.0042 (14)	−0.0012 (12)	−0.0061 (12)
O23	0.0381 (15)	0.0328 (16)	0.0419 (16)	0.0107 (13)	−0.0008 (12)	0.0005 (13)
C23	0.065 (3)	0.042 (3)	0.052 (3)	0.013 (2)	−0.014 (2)	0.002 (2)
C1A	0.039 (2)	0.032 (2)	0.037 (2)	−0.0049 (19)	0.0036 (17)	0.0032 (18)
C2A	0.060 (3)	0.037 (2)	0.041 (2)	−0.006 (2)	0.015 (2)	0.006 (2)
C3A	0.073 (3)	0.043 (3)	0.024 (2)	0.004 (2)	0.002 (2)	0.0043 (19)
C4A	0.053 (3)	0.049 (3)	0.033 (2)	0.003 (2)	−0.0079 (19)	−0.002 (2)
C5A	0.035 (2)	0.033 (2)	0.034 (2)	−0.0004 (18)	0.0001 (16)	−0.0016 (17)
C6A	0.0329 (19)	0.0182 (17)	0.0267 (18)	0.0032 (15)	0.0028 (14)	−0.0003 (14)
C7	0.0192 (16)	0.0193 (17)	0.0286 (17)	−0.0045 (14)	0.0022 (13)	0.0002 (14)
O7	0.0219 (12)	0.0242 (13)	0.0341 (14)	−0.0034 (11)	0.0015 (10)	0.0061 (11)
O62	0.012 (2)	0.016 (3)	0.029 (3)	−0.0013 (18)	−0.0002 (16)	−0.0083 (19)
C61	0.094 (14)	0.066 (16)	0.057 (10)	0.005 (12)	0.004 (9)	0.026 (10)
O72	0.035 (4)	0.031 (4)	0.046 (4)	−0.006 (3)	−0.003 (3)	−0.003 (3)
C71	0.043 (9)	0.054 (15)	0.105 (15)	−0.015 (10)	0.011 (9)	−0.003 (13)

### Geometric parameters (Å, °)

**Table:**

P1—O12	1.478 (3)	C23—H23F	0.9600
P1—O13	1.548 (3)	C1A—C2A	1.388 (6)
P1—O11	1.551 (3)	C1A—C6A	1.396 (5)
P1—C7	1.851 (3)	C1A—H1A	0.9300
O11—H11	0.8200	C2A—C3A	1.379 (7)
O13—H13	0.8200	C2A—H2A	0.9300
P2—O21	1.489 (3)	C3A—C4A	1.376 (6)
P2—O22	1.507 (3)	C3A—H3A	0.9300
P2—O23	1.575 (3)	C4A—C5A	1.389 (6)
P2—C7	1.857 (4)	C4A—H4A	0.9300
O21—H21	0.8200	C5A—C6A	1.389 (5)
O22—H22	0.8200	C5A—H5A	0.9300
O23—C23	1.457 (5)	C6A—C7	1.534 (5)
C23—H23A	0.9600	C7—O7	1.426 (4)
C23—H23B	0.9600	O7—H7	0.8200
C23—H23C	0.9600	O62—C61	1.384 (18)
C23—H23D	0.9600	O72—C71	1.37 (2)
C23—H23E	0.9600		
			
O12—P1—O13	113.27 (16)	O23—C23—H23F	109.5
O12—P1—O11	113.79 (16)	H23A—C23—H23F	56.3
O13—P1—O11	106.55 (15)	H23B—C23—H23F	56.3
O12—P1—C7	110.81 (16)	H23C—C23—H23F	141.1
O13—P1—C7	105.00 (15)	H23D—C23—H23F	109.5
O11—P1—C7	106.82 (15)	H23E—C23—H23F	109.5
P1—O11—H11	109.5	C2A—C1A—C6A	120.4 (4)
P1—O13—H13	109.5	C2A—C1A—H1A	119.8
O21—P2—O22	117.32 (17)	C6A—C1A—H1A	119.8
O21—P2—O23	111.97 (16)	C3A—C2A—C1A	120.4 (4)
O22—P2—O23	103.58 (16)	C3A—C2A—H2A	119.8
O21—P2—C7	111.78 (16)	C1A—C2A—H2A	119.8
O22—P2—C7	107.03 (16)	C4A—C3A—C2A	119.5 (4)
O23—P2—C7	104.03 (15)	C4A—C3A—H3A	120.3
P2—O21—H21	109.5	C2A—C3A—H3A	120.3
P2—O22—H22	109.5	C3A—C4A—C5A	120.9 (4)
C23—O23—P2	125.2 (3)	C3A—C4A—H4A	119.6
O23—C23—H23A	109.5	C5A—C4A—H4A	119.6
O23—C23—H23B	109.5	C6A—C5A—C4A	120.0 (4)
H23A—C23—H23B	109.5	C6A—C5A—H5A	120.0
O23—C23—H23C	109.5	C4A—C5A—H5A	120.0
H23A—C23—H23C	109.5	C5A—C6A—C1A	118.9 (3)
H23B—C23—H23C	109.5	C5A—C6A—C7	119.5 (3)
O23—C23—H23D	109.5	C1A—C6A—C7	121.5 (3)
H23A—C23—H23D	141.1	O7—C7—C6A	112.6 (3)
H23B—C23—H23D	56.3	O7—C7—P1	103.2 (2)
H23C—C23—H23D	56.3	C6A—C7—P1	111.2 (2)
O23—C23—H23E	109.5	O7—C7—P2	108.1 (2)
H23A—C23—H23E	56.3	C6A—C7—P2	109.0 (2)
H23B—C23—H23E	141.1	P1—C7—P2	112.63 (17)
H23C—C23—H23E	56.3	C7—O7—H7	109.5
H23D—C23—H23E	109.5		
			
O21—P2—O23—C23	−32.5 (4)	O13—P1—C7—O7	−66.9 (2)
O22—P2—O23—C23	−159.8 (3)	O11—P1—C7—O7	46.0 (2)
C7—P2—O23—C23	88.4 (3)	O12—P1—C7—C6A	−68.5 (3)
C6A—C1A—C2A—C3A	−0.9 (7)	O13—P1—C7—C6A	54.1 (3)
C1A—C2A—C3A—C4A	1.5 (7)	O11—P1—C7—C6A	167.0 (2)
C2A—C3A—C4A—C5A	−0.4 (7)	O12—P1—C7—P2	54.2 (2)
C3A—C4A—C5A—C6A	−1.3 (7)	O13—P1—C7—P2	176.81 (17)
C4A—C5A—C6A—C1A	1.9 (6)	O11—P1—C7—P2	−70.3 (2)
C4A—C5A—C6A—C7	−174.2 (4)	O21—P2—C7—O7	−171.4 (2)
C2A—C1A—C6A—C5A	−0.8 (6)	O22—P2—C7—O7	−41.7 (3)
C2A—C1A—C6A—C7	175.2 (4)	O23—P2—C7—O7	67.6 (2)
C5A—C6A—C7—O7	−14.6 (5)	O21—P2—C7—C6A	65.9 (3)
C1A—C6A—C7—O7	169.4 (3)	O22—P2—C7—C6A	−164.4 (2)
C5A—C6A—C7—P1	−129.9 (3)	O23—P2—C7—C6A	−55.1 (3)
C1A—C6A—C7—P1	54.1 (4)	O21—P2—C7—P1	−58.0 (2)
C5A—C6A—C7—P2	105.4 (3)	O22—P2—C7—P1	71.7 (2)
C1A—C6A—C7—P2	−70.6 (4)	O23—P2—C7—P1	−179.03 (17)
O12—P1—C7—O7	170.5 (2)		

### Hydrogen-bond geometry (Å, °)

**Table:**

*D*—H···*A*	*D*—H	H···*A*	*D*···*A*	*D*—H···*A*
O13—H13···O21^i^	0.82	1.75	2.548 (4)	163.
O11—H11···O22^ii^	0.82	1.72	2.525 (4)	169.
O21—H21···O62^iii^	0.82	1.89	2.548 (5)	136.
O7—H7···O12^iv^	0.82	1.86	2.658 (3)	164.

Symmetry codes: (i) *x*, *y*+1, *z*; (ii) −*x*+1, −*y*, −*z*; (iii) *x*, *y*−1, *z*; (iv) *x*+1, *y*, *z*.
